# Supplementary material for: DNA Methylation of Window of Implantation Genes in Cervical Secretions Predicts Ongoing Pregnancy in Infertility Treatment
Source: Int J Mol Sci. 2023 Mar 15;24(6):5598. doi: 10.3390/ijms24065598 (PMC10051225; doi:10.3390/ijms24065598)
Supplement: Supplementary file 1 [file ijms-24-05598-s001.zip › ijms-2215309-supplementary/Supplementary figures.pdf]

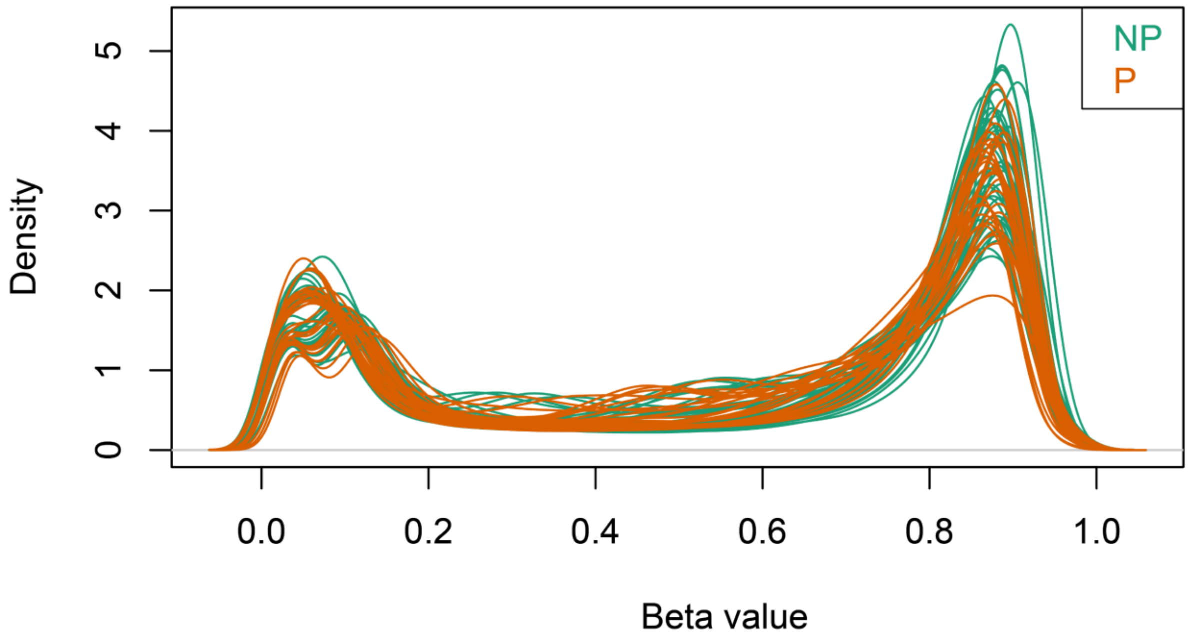

Figure S1. Density plot of cervical secretion genome-wide methylation profiles. Genome-wide methylation profiles measured by an Illumina EPIC 850K BeadChip array from 31 pregnancy and 37 nonpregnancy cervical secretion samples. Density plot demonstrates the distribution of β-values measured at each of the 743,730 DNA methylation probes. Values from either probe type (I and II) on this platform were normalized by the BMIQ method.

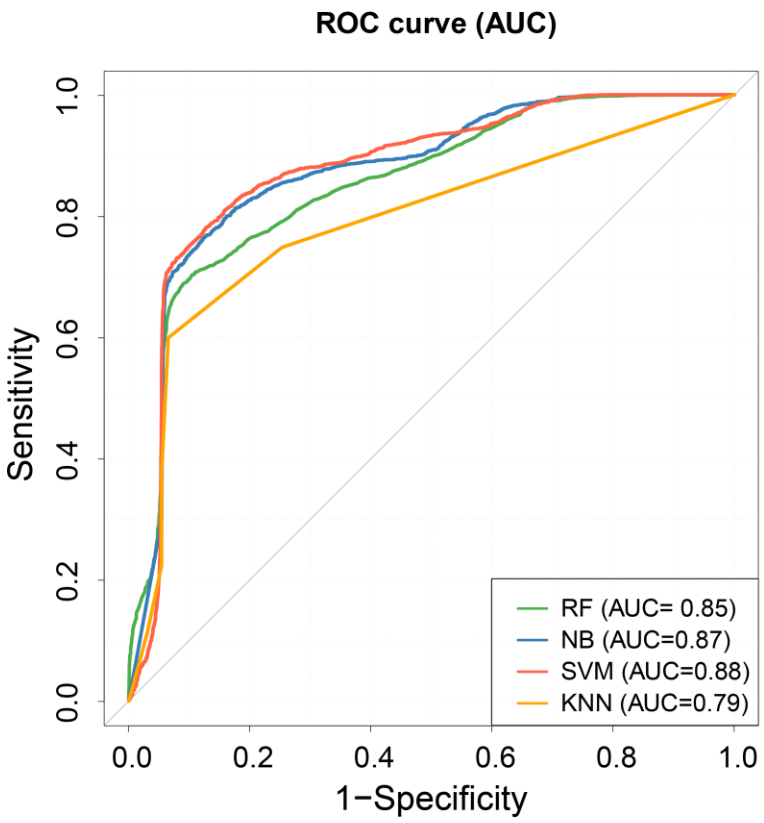

Figure S2. AUCs of four ML algorithms using 152 DMPs for predicting ongoing pregnancy. ROC curves (AUCs) of the best models from four ML algorithms using 152 DMPs for predicting ongoing pregnancy in the array dataset. AUC values were generated from a 100–time repeated fivefold cross-validation. ML: machine learning, DMP: differentially methylated probe, ROC: receiver operating characteristic, AUC: area under the ROC curve, RF: random forest, NB: naïve Bayes, SVM: support vector machine, KNN: k-nearest neighbor.

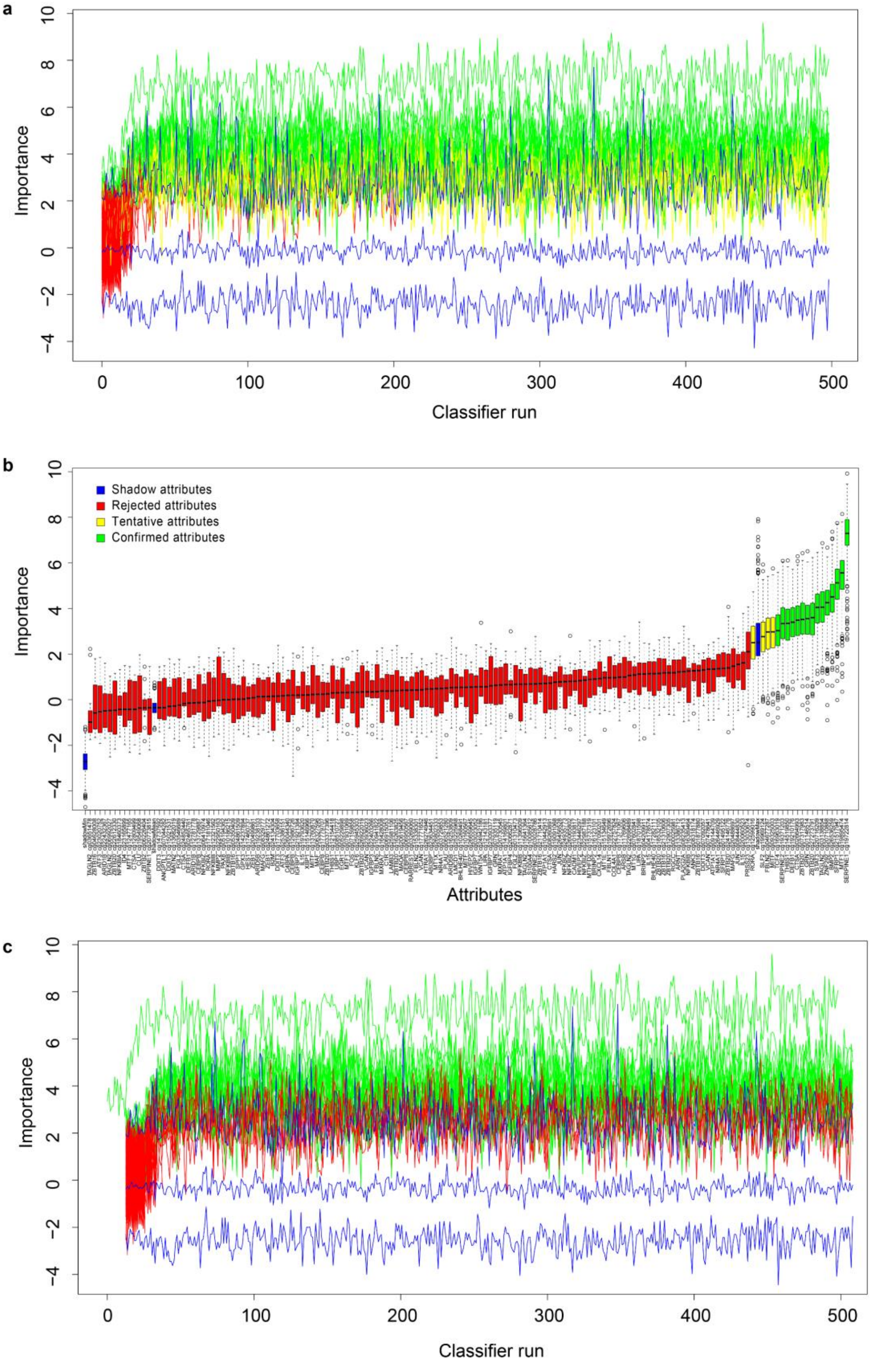
a
Importance
Classifier run
b
Shadow attributes
Rejected attributes
Tentative attributes
Confirmed attributes
Importance
Attributes
c
Importance
Classifier run

Figure S3. Classifier run history and importance ranking of 152 attributes (DMPs) by the Boruta algorithm. (**a**). Importance values of all attributes in each classifier run before tentative fix. (**b**). Importance ranking of 152 attributes (DMPs) compared with shadow attributes by BA before tentative fix. Blue, red, yellow, and green box plots represent the importance levels of the shadow, rejected, tentative, and confirmed attributes, respectively. (**c**). Importance values of all attributes in each classifier run after tentative fix.

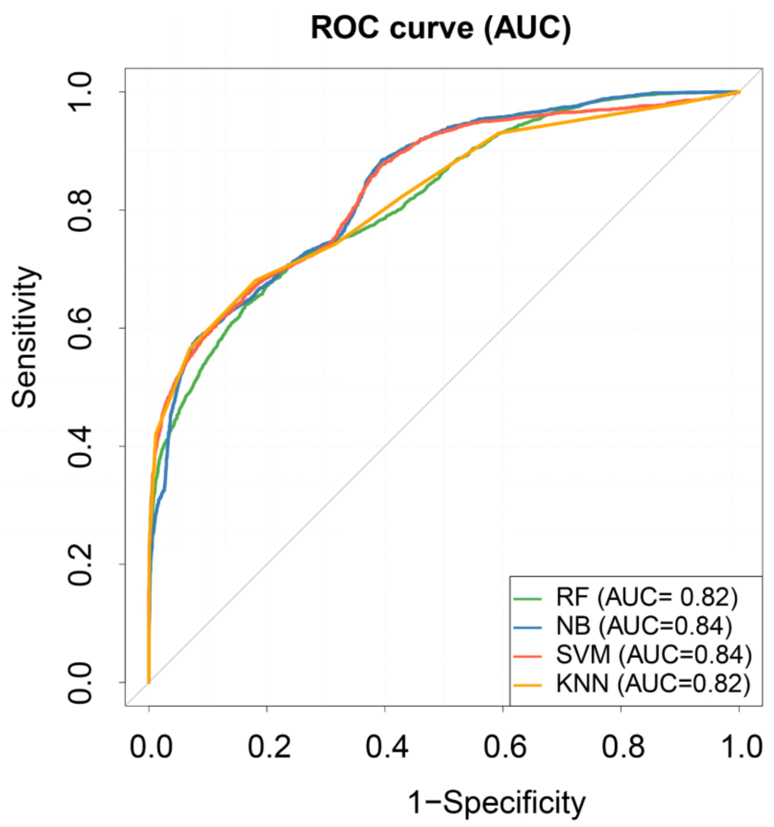

Figure S4. AUCs of four ML algorithms using three DMPs for predicting ongoing pregnancy. ROC curves (AUCs) of the best models from four ML algorithms using three selected DMPs from three candidate genes for predicting ongoing pregnancy in the array dataset. The AUC values were generated from a 100–time repeated fivefold cross-validation. The DMPs include cg19722814

(SERPINE1), cg19371203 (SERPINE2), and cg03138854 (TAGLN2). ML: machine learning, DMP: differentially methylated probe, ROC: receiver operating characteristic, AUC: area under the ROC curve, RF: random forest, NB: naïve Bayes, SVM: support vector machine, KNN: k-nearest neighbor.
